# Combined  Application of a Composite Microbial Inoculant and Bulking Agents Improves Chicken Manure Composting

**DOI:** 10.3390/microorganisms14092052

**Published:** 2026-09-14

**Authors:** Junhuan Gao, Dongying Zhao, Yibing Zhao, Fei Liu, Haofang Zhu, Zanxia Cao, Jihua Wang, Zelin Li, Ziyang Li, Linshuang Hu, Zhenghua Li

**Affiliations:** 1 Institute of Biophysics, Dezhou University, Dezhou 253023, China; 16652156821@163.com (J.G.); zhaodongying4321@163.com (D.Z.); 17615931631@163.com (F.L.); zhuh1360@gmail.com (H.Z.); jhw25336@126.com (J.W.); 15264102340@163.com (Z.L.); 15689889855@163.com (Z.L.); 2 School of Life Sciences, Dezhou University, Dezhou 253023, China; qiayilai@mail.ustc.edu.cn; 3 Department of Intelligent Manufacturing, Dezhou Vocational and Technical College, Dezhou 253034, China

**Keywords:** chicken manure, aerobic composting, composite microbial inoculant, bulking agents of plant origin, odorous compounds, germination index

## Abstract

Intensive poultry farming produces substantial volumes of chicken manure that require effective and hygienic management. This study investigated a composite inoculant formulated with four microorganisms: *Kocuria rosea*  ACCC 10488, *Bacillus subtilis* ACCC 60364, *Trichoderma longibrachiatum* ACCC 32584, and *Lactobacillus plantarum* ACCC 11118. This study investigated a composite inoculant formulated with four microorganisms obtained from the Agricultural Culture Collection of China (ACCC): *Kocuria rosea* ACCC 10488, *Bacillus subtilis* ACCC 60364, *Trichoderma longibrachiatum* ACCC 32584, and *Lactobacillus plantarum* ACCC 11118. Its performance was examined both without amendment and after addition of plant-derived bulking materials. The experiment included five treatments: untreated chicken manure (T1), the bulking-agent mixture without microbial addition (T2), a commercial inoculant combined with the same bulking materials (T3), the newly prepared inoculant without bulking agents (T4), and the newly prepared inoculant combined with the bulking mixture (T5). Three independent 50-kg piles were prepared for every treatment. Temperature was recorded for 45 days; physicochemical characteristics, odor indicators, nutrient concentrations, enzyme activities, seed-bioassay responses, and Ascaris egg mortality were evaluated over the initial 15 days. T5 reached 74 °C on days 5 and 10 and remained above 50 °C through day 30 at the plotted observation points. On day 15, this treatment had lower measured levels of odorous compounds, greater concentrations of available phosphorus and potassium, a 99% germination index, and complete mortality of the assayed Ascaris eggs. Collectively, T5 showed favorable changes across several indicators of early composting performance under the conditions tested. The study did not examine microbial community structure, persistence of the introduced strains, functional genes, whole-pile nutrient mass balances, or longer-term agronomic performance. Consequently, the data neither establish synergistic interactions among the component strains nor identify specific nutrient-transformation pathways.

## 1. Introduction

The continued intensification and industrialization of poultry production, particularly in China, generate large quantities of chicken manure each year [1]. Fresh chicken manure contains abundant organic matter and mineral nutrients, including nitrogen, phosphorus, and potassium. It therefore has considerable potential as a renewable organic resource for agricultural production [2]. Nevertheless, untreated chicken manure may also contain high loads of pathogenic microorganisms, parasite eggs, and antibiotic resistance genes [3]. Consequently, its direct application to agricultural soils may cause salinization, damage crop roots, and facilitate the transmission of diseases through soil [3,4]. The uncontrolled stockpiling of raw chicken manure creates additional environmental and health concerns because it releases large amounts of odorous pollutants, particularly volatile organic compounds (VOCs), ammonia, and hydrogen sulfide. These emissions can substantially degrade air quality and threaten the health of humans and livestock [5].

Bioaugmentation and physical amendment are both used to improve livestock manure composting. Functional inoculants may provide hydrolytic, acidifying, and other metabolic functions that mobilize nutrients, whereas lignocellulosic bulking agents can modify porosity, moisture distribution, and the carbon-to-nitrogen C/N characteristics of the substrate [6,7,8]. However, few studies have compared a defined composite inoculant, used alone or with bulking agents, with both bulking agents alone and a commercial inoculant under identical conditions. It therefore remains unclear whether combining microbial inoculation with a defined mixture of bulking agents improves composting during its early stages. Specifically, it is uncertain whether the combination outperforms either component alone.

To address this gap, we prepared a composite inoculant comprising *Kocuria rosea*, *Bacillus subtilis*, *Trichoderma longibrachiatum*, and *Lactobacillus plantarum*. The treatment design distinguished the effects of the inoculant prepared in this study and the bulking agent mixture and included a commercial inoculant benchmark under the same amended conditions. The study compared temperature, odor indicators, nutrient concentrations, enzyme activities, phytotoxicity in a seed bioassay, and Ascaris egg mortality. The objective was to determine whether the combined treatment improved these endpoints during the first 15 days of composting.

## 2. Materials and Methods

### 2.1. Composting Materials

Chicken manure was obtained from a local poultry farm in Decheng District, Dezhou City, Shandong Province, China. Corn straw was purchased from Xinde Agricultural Products Co., Ltd. (Lingshou County, Shijiazhuang, China). It was cut into pieces approximately 3 cm long [9] and then combined with furfural residue derived from corncobs and plant ash. These locally available materials of plant origin were selected as bulking amendments intended to improve matrix structure and provide carbonaceous and mineral components. Their principal physicochemical characteristics are presented in Table 1. To characterize the raw materials, one homogenized sample of each material was analyzed in triplicate; these were analytical replicates rather than additional independent composting units.

Four functional microbial strains  were used to prepare the inoculant: *K*. *rosea* ACCC 10488, *B*. *subtilis* ACCC 60364, *T*. *longibrachiatum* ACCC 32584, and *L*. *plantarum* ACCC 11118. All four strains were obtained from the Agricultural Culture Collection of China (ACCC), Beijing, China. *T. longibrachiatum *was cultivated on potato dextrose agar (PDA), whereas *K. rosea *and *B. subtilis *were cultivated in Luria–Bertani (LB) medium.*L. plantarum *was cultivated in de Man, Rogosa, and Sharpe (MRS) medium.

The solid fermentation medium was prepared as described previously [10]. It contained 0.8% glucose, 36.4% bran, 54% barley residue, 8% corn flour, 0.5% calcium carbonate, 0.16% potassium dihydrogen phosphate, and 0.14% sodium chloride. Water was added to adjust the moisture content, and the pH was adjusted to 6.5–7.0. This medium was used for solid-state fermentation of the composite microbial inoculant.

### 2.2. Preparation of the Composite Inoculant

Seed broths of *K. rosea*, *B. subtilis*, *T. longibrachiatum*, and *L. plantarum* were combined at a volume ratio of 1:4:2.5:2.5. The resulting composite seed culture was introduced into the solid fermentation medium at an inoculation rate of 4% (*w*/*w*). Solid-state fermentation was then performed at 30 °C while maintaining the moisture content at 60% [11]. The fermentation process was terminated once the viable cell count in the fermented material exceeded 1 × 10^8^ colony-forming units per gram (CFU g^−1^). The resulting fermentation product was then air-dried to a moisture content of 25% (*w*/*w*), yielding the final composite microbial inoculant [12].

### 2.3. Composting Design

Five treatments were established for predefined comparisons. T1 contained chicken manure only and served as the unamended baseline. T2 contained chicken manure plus corn straw, furfural residue, and plant ash and was used to assess the effect of matrix amendment without inoculation. T3 used the same bulking agent mixture as T2 plus a commercial compost inoculant and served as a benchmark for the commercial inoculant. T4 contained chicken manure and the composite inoculant prepared in this study but no bulking agents. This treatment was used to assess inoculant performance in the unamended manure matrix. T5 combined chicken manure, the three bulking agents, and the composite inoculant prepared in this study. Comparisons of T5 with T2 and T4 indicate the performance of the combined treatment relative to either component treatment. The comparison of T5 with T3 evaluates the inoculant prepared in this study against the commercial product in the same amended matrix. This design supports comparisons among treatments but cannot establish microbial synergy without a formal interaction analysis or additional mechanistic controls. Each pile had a total mass of 50 kg. Three independently prepared piles were used per treatment (*n* = 3), giving 15 piles in total.

All material ratios were calculated on a fresh mass basis. T2 contained chicken manure, corn straw, furfural residue, and plant ash at a ratio of 850:50:50:50. T3 used the same mixture of four components with the commercial inoculant at a ratio of 850:50:50:50:1. T4 contained chicken manure and the composite inoculant prepared in this study at a ratio of 850:1. T5 contained chicken manure, corn straw, furfural residue, plant ash, and the composite inoculant prepared in this study at a ratio of 850:50:50:50:1. These rates corresponded to approximately 1.00 g kg^−1^ of the final fresh mixture in T3 and T5 and 1.18 g kg^−1^ in T4. The commercial inoculant used in T3 was supplied by Zhengzhou Haowangnong Biotechnology Co., Ltd. (Zhengzhou, China; catalog no. SWJFFJJ). The experimental record listed *Bacillus licheniformis*, *Enterococcus faecalis*, lactic acid bacteria, and yeasts as its principal microbial components and reported a total viable count of 4.5 × 10^8^ CFU g^−1^.

Before composting, each pile had an initial mass of 50 kg. Its moisture content was adjusted to 60%, and the pile was covered with plastic film. During the first 15 days, the film was removed every 2 days for at least 2 h of natural aeration, and each pile was turned thoroughly during the aeration period. From day 16 to day 45, each pile was manually turned once every 5 days to redistribute the material and renew passive aeration; no forced aeration system was used [13]. Temperature was recorded daily throughout the observation period of 45 days. Samples for the other endpoints were collected on days 0, 5, 10, and 15. At each sampling time, five subsamples from each independent pile were combined and homogenized to obtain an approximately 500 g composite sample representative of each pile [14].

### 2.4. Determination of Indicators and Analytical Methods

The three independently prepared compost piles within each treatment served as the experimental replicates (*n* = 3). At each sampling time, the material collected from five points within each pile was combined and homogenized to form one composite sample for that pile. When an assay was performed in triplicate on the same composite sample, the measurements were treated as technical replicates. They were averaged to produce one value for the pile and were not counted as additional independent observations. The three values, one for each pile, were used to calculate treatment means and variability and to compare treatments.

Available phosphorus was quantified using the molybdenum–antimony colorimetric method, whereas available potassium was measured by flame photometry [15]. The temperature at the center of every compost pile was monitored daily at 8:30 a.m. throughout the composting period. Measurements were obtained at a depth of 20 cm using an electronic thermometer, and the corresponding ambient temperature was recorded at the same time [16].

For the determination of pH and electrical conductivity (EC), fresh compost was mixed with deionized water at a ratio of 1:10 (*w*/*v*) [17]. The suspensions were agitated at 180 rpm for 1 h, after which the supernatants were collected. The pH and EC of each supernatant were then determined using a calibrated combined pH/EC meter [18].

Moisture content (MC), organic matter content, and total nitrogen were determined using 10.00 g portions of compost. The samples were dried to constant mass in an oven with forced air circulation at 105 °C. MC was calculated from the change in sample mass before and after drying [19]. Organic matter content was measured using the potassium dichromate volumetric method [20], whereas total nitrogen was determined using the Kjeldahl method [21].

Organic carbon was not measured independently. The potassium dichromate volumetric method reports organic matter by applying the conventional factor 1.724 to the organic carbon equivalent [22]. Therefore, the organic carbon mass fraction was calculated from the reported organic matter value as follows:

(1) *w*_OC_ = *w*_OM_/1.724 ≈ 0.5800 *w*_OM_

The operational C/N ratio was calculated as follows:

(2) C/N = *w*_OC_/*w*_TN_

where *w*_OC_, *w*_OM_, and *w*_TN_ denote the dry mass percentages of organic carbon, organic matter, and total nitrogen, respectively. Because organic carbon was derived using the conversion factor specific to this analytical method rather than measured independently by elemental combustion, the resulting C/N values represent operational OC/TN estimates. The factor 1.724 is a conventional conversion factor.

NH_3_ and H_2_S concentrations were measured directly in the pile gas using a GasAlertMicro 5 portable multi-gas detector (BW Technologies by Honeywell, Mississauga, ON, Canada) equipped with electrochemical sensors and an integral motorized sampling pump. Measurements were performed on days 0, 5, 10, and 15 within the same daily time window and before pile turning to minimize variation caused by operational disturbance [23]. For each independent pile, gas was sampled from five fixed positions comprising the pile center and four evenly distributed peripheral positions, with the sampling inlet maintained approximately 10 cm above the pile surface. At each position, the detector was operated for 60 s, and three consecutive readings were recorded after the signal had stabilized. The sampling line was flushed with clean air between piles until the detector returned to baseline. Readings from the five positions were averaged to obtain one representative value for each pile. The three independently prepared piles per treatment were regarded as biological replicates (*n* = 3), whereas repeated readings within each pile were treated as technical measurements. Concentrations were recorded directly in the instrument-displayed unit of mg L^−1^.

To determine the seed germination index (GI) and vigor index (VI), fresh compost samples were extracted with deionized water at a ratio of 1:10 (*w*/*v*). The mixtures were shaken at 100 rpm for 1 h at 25 °C and subsequently passed through qualitative filter paper. An aliquot (5 mL) of each filtrate was added to a Petri dish lined with sterile filter paper. Ten fully developed pak choi seeds were then distributed evenly across the paper surface. The dishes were incubated in darkness at 25 ± 2 °C for 48 h. Following incubation, the number of germinated seeds and the length of the primary root were recorded [24]. Deionized water served as the blank control. The GI was calculated according to the following equation [25]:

(3) GI (%) = [(*G_t_* × *L_t_*)/(*G_c_* × *L_c_*)] × 100

In Equation (3), *G_t_* and *L_t_* denote the germination percentage and mean root length, respectively, in the compost extract. *G_c_* and *L*_c_ denote the corresponding values in the deionized water control.

After measuring root length, all germinated seedlings from each Petri dish were collected and gently blotted with filter paper. They were then dried in an oven with forced air circulation at 65 °C to constant mass. The dried seedlings were cooled in a desiccator and weighed. Mean seedling dry mass (S, mg seedling^−1^) was calculated by dividing the total seedling dry mass in each dish by the number of germinated seedlings.

The vigor index was subsequently calculated as follows:

(4) VI = *S* × GI

Note: S denotes the mean seedling dry mass per germinated seedling (mg seedling^−1^).

The mortality of Ascaris eggs was determined according to GB/T 19524.2-2004 [26]. Eggs were separated from the homogenized compost samples by alkaline dispersion, recovered by saturated sodium chloride flotation, and subjected to microscopic viability assessment following the staining and culture procedures specified in the standard. Mortality was calculated as the number of non-viable eggs divided by the total number of viable and non-viable eggs examined and expressed as a percentage [27]. The resulting mortality was evaluated against the requirement specified for organic fertilizers in NY/T 525-2021 [28], which requires an Ascaris egg mortality of ≥95%. The three independently prepared piles per treatment were treated as biological replicates (*n* = 3), and repeated microscopic counts from each pile were averaged.

Phosphate solubilization by the strains was assessed by measuring the clear zones formed on National Botanical Research Institute’s phosphate growth medium (NBRIP) and organic phosphorus agar. Potassium solubilization was assessed using Aleksandrov medium. The available potassium concentration in the resulting culture solution was determined by flame photometry and used to calculate the potassium solubilization rate [29].

Potential antagonistic interactions among the four strains were examined by the cross-streak method on PDA solid medium. Following incubation at 30 °C for 24 h, the resulting microbial growth patterns were visually examined and recorded [30].

### 2.5. Enzyme Activity Assays

Each fresh compost sample was separated into two subsamples immediately after collection. One subsample was stored at 4 °C and analyzed for enzyme activity within 72 h to minimize irreversible activity loss during air drying. The second subsample was air-dried at room temperature, finely ground, and passed through a 250 μm sieve before analysis of the basic physicochemical properties of the compost. The activities of four key enzymes were subsequently measured. Cellulase activity was quantified by the 3,5-dinitrosalicylic acid (DNS) colorimetric method using sodium carboxymethyl cellulose (CMC-Na) as the reaction substrate [31]. Hemicellulase activity was assessed using the same DNS colorimetric method, with xylan serving as the specific substrate [32]. Urease (EC 3.5.1.5) activity was measured using the phenol–sodium hypochlorite colorimetric method. Activity was calculated from the amount of ammonium nitrogen (NH_4_^+^-N) released during urea hydrolysis [33]. Alkaline phosphatase (EC 3.1.3.1) activity was determined using the sodium phenyl phosphate colorimetric method in borate buffer adjusted to pH 9.6 [34,35]. Enzyme activities were expressed as enzyme units per gram of dry weight (U g^−1^ DW).

### 2.6. Data Analysis

Microsoft Excel 2016 was used for preliminary data organization, IBM SPSS Statistics 26.0 was used for statistical analyses, and Origin 2018 was used to prepare the figures. The independently prepared compost pile was the experimental unit (*n* = 3 per treatment). Triplicate technical measurements of the same homogenized sample from each pile were averaged before inferential analysis. Data are presented as mean ± standard deviation SD of the three independent piles unless otherwise stated. Treatment means were compared separately at each sampling time using one-way analysis of variance (ANOVA). When the omnibus ANOVA indicated a treatment effect, Duncan’s multiple range test was used for post hoc grouping. The in vitro strain assays were analyzed separately using the same statistical approach. Statistical significance was defined as *p* < 0.05. Within a table column or sampling time, means sharing at least one lowercase letter were not significantly different under Duncan’s procedure. Because each sampling time was analyzed separately, no overall time effect or interaction between treatment and time was inferred from these tests.

## 3. Results

### 3.1. Antagonistic Interactions and Functional Characteristics of the Composite Strains

All four strains grew at the intersections of the crossed streaks without visible inhibition under the tested conditions (Figure S1). The assay therefore detected no antagonism among *K. rosea*, *B. subtilis*, *T. longibrachiatum*, and *L. plantarum* in vitro. It did not test for beneficial interactions, persistence, or coexistence in the compost matrix.

The individual strains and the composite culture formed measurable dissolution zones and solubilized potassium in vitro (Table 2). Among the tested cultures, the composite culture produced the largest dissolution zone (92.4 ± 2.5 mm) and showed the highest potassium solubilization efficiency (96.5 ± 1.1%).

### 3.2. Temperature Dynamics During Chicken Manure Composting

**Figure 1:**
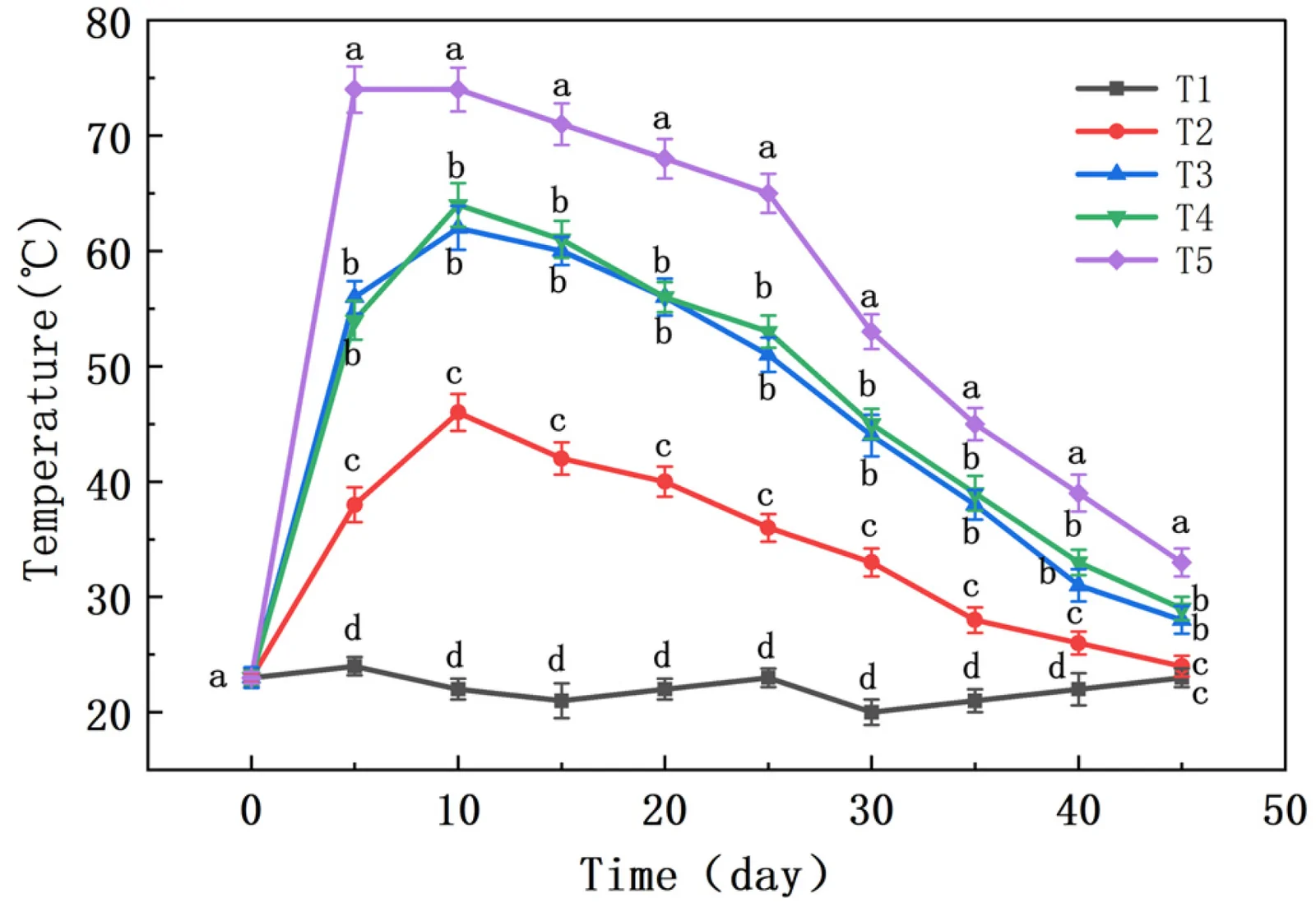
Temperature dynamics of the five treatments throughout the 45 days of observation. T1, chicken manure only; T2, chicken manure with corn straw, furfural residue, and plant ash; T3, the T2 formulation supplemented with the commercial inoculant; T4, chicken manure supplemented with the composite inoculant but without bulking agents; and T5, the T2 formulation supplemented with the composite inoculant. Data are presented as mean ± SD of three independently prepared compost piles (*n* = 3). At each sampling time, different lowercase letters indicate significant differences among treatments according to one-way ANOVA followed by Duncan’s multiple range test (*p* < 0.05); treatments sharing at least one letter are not significantly different. The single ‘a’ at day 0 denotes a shared grouping for all five treatments.

Temperature is an important indicator of microbial metabolic activity and the progression of compost maturation [36]. The temperature profiles of the five treatments over 45 days of composting are presented in Figure 1.

T1 remained at 20–24 °C throughout the observation period and did not develop a clear heating phase (Figure 1). T2 reached a maximum of 46 °C on day 10. T3 and T4 reached maxima of 62 °C and 64 °C, respectively, on day 10. T5 heated most rapidly, reached 74 °C on days 5 and 10, and remained above 50 °C through day 30 at the plotted sampling times.

Across the plotted time points, T5 showed the greatest thermal response, followed by T3 and T4, whereas T1 showed little heating. These statements describe the observed profiles. Accurate durations above 40 °C or 50 °C cannot be determined from the plotted time points and should instead be calculated from the original daily records for each pile.

### 3.3. Changes in the C/N Ratio

**Figure 2:**
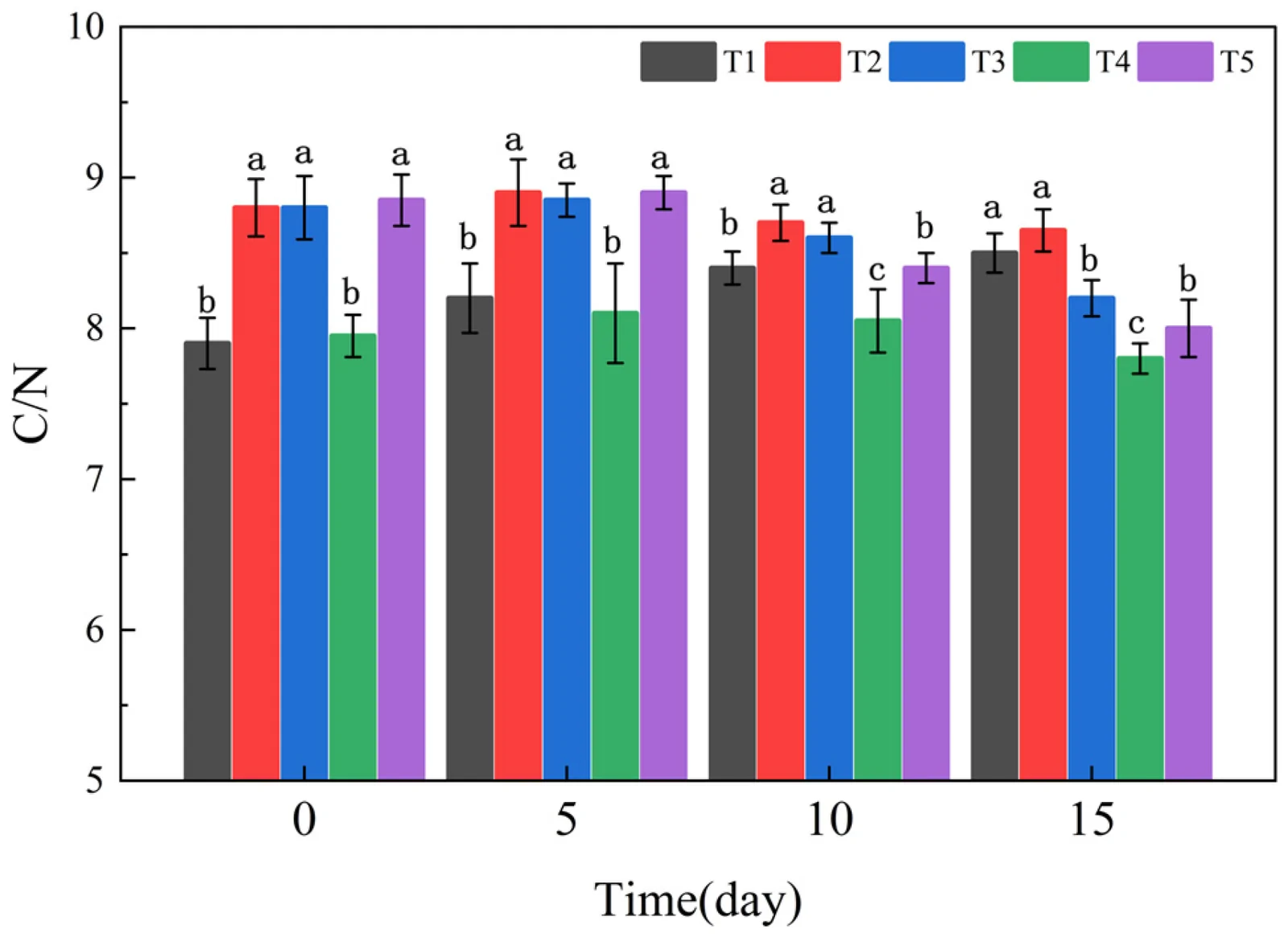
Carbon-to-nitrogen (C/N) ratios of the five treatments during the first 15 days of composting. Data are mean ± SD of three independently prepared compost piles (*n* = 3). Different lowercase letters at the same sampling time indicate significant differences according to one-way ANOVA followed by Duncan’s multiple range test (*p* < 0.05); treatments sharing at least one letter are not significantly different.

The changes in the C/N ratios of the five treatments during composting are shown in Figure 2. At day 15, T1 and T2 had C/N ratios of 8.50 and 8.65, respectively, with no significant difference between them (*p* > 0.05). T3 and T5 had ratios of 8.20 and 8.00, respectively. These values did not differ significantly from each other (*p* > 0.05), but both were significantly lower than those of T1 and T2 (*p* < 0.05). T4 exhibited the lowest C/N ratio (7.80 ± 0.10), which was significantly lower than those of all other treatments (*p* < 0.05).

### 3.4. Changes in Odor Indicators

**Figure 3:**
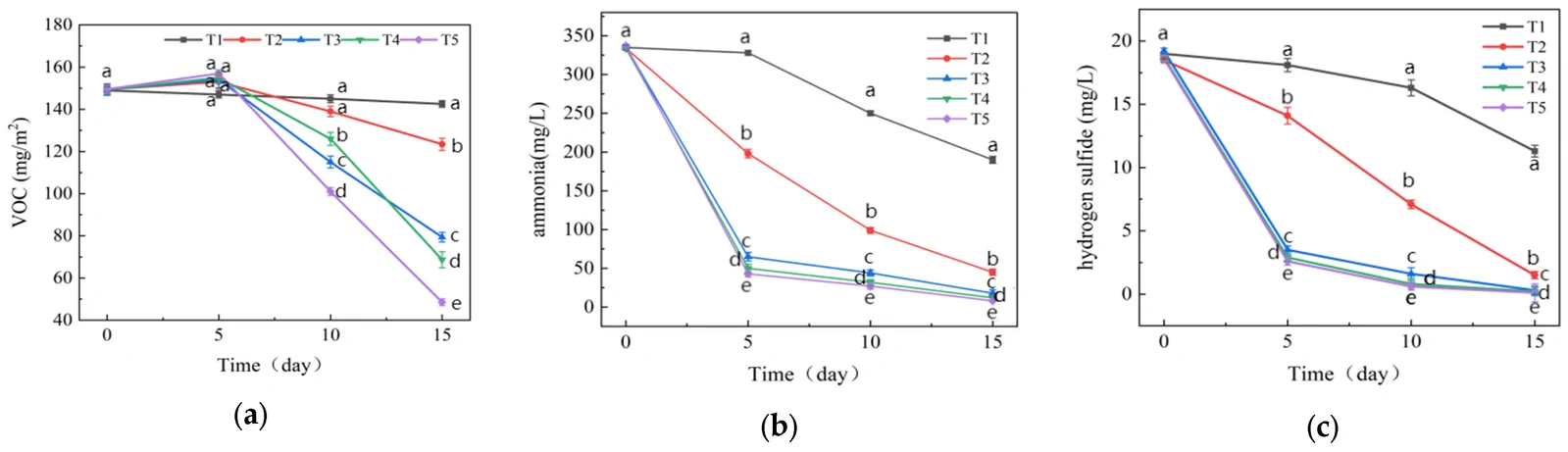
Odor indicators during the first 15 days of chicken manure composting: (**a**) the reported volatile organic compound (VOC) endpoint; (**b**) ammonia; and (**c**) hydrogen sulfide. Data are presented as mean ± SD of three independently prepared compost piles (*n* = 3). Different lowercase letters at the same sampling time indicate significant differences according to one-way ANOVA followed by Duncan’s multiple range test (*p* < 0.05); treatments sharing at least one letter are not significantly different. On day 0, the single letter “a” applies collectively to all five treatments because no significant differences were detected among them.

After 15 days of composting, T1 showed a VOC removal rate of only 3.88%. By comparison, the removal rate in T5 reached 67.23% and was significantly higher than the rates in all other treatments (*p* < 0.05; Figure 3a). The temporal changes in ammonia and hydrogen sulfide concentrations are presented in Figure 3b and Figure 3c, respectively. In T1 and T2, the concentrations of both gases decreased slowly, and a strong odor remained detectable on day 15. By contrast, the measured odor indicators in T4 and T5 decreased substantially by day 10 and remained low on day 15. In T5, the ammonia concentration decreased from 330.42 mg/L to 10.07 mg/L after 15 days, corresponding to a reduction of 96.95%. Over the same period, the hydrogen sulfide concentration declined from 18.46 mg/L to 0.018 mg/L, representing a reduction of 99.90%. On day 15, the measured ammonia and hydrogen sulfide concentrations in T5 were significantly lower than the corresponding concentrations in T1 (*p* < 0.05).

### 3.5. Changes in Nutrient Concentrations

**Figure 4:**
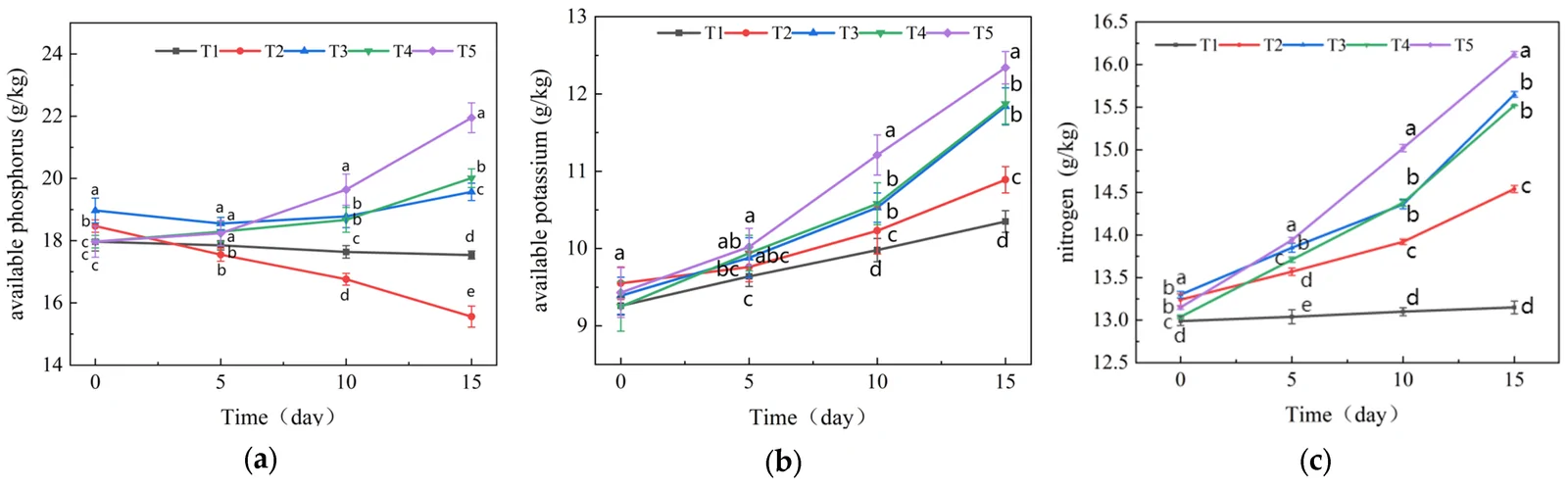
Nutrient concentrations during the first 15 days of composting: (**a**) available phosphorus; (**b**) available potassium; and (**c**) total nitrogen. Data are presented as mean ± SD of three independently prepared compost piles (*n* = 3). Different lowercase letters at the same sampling time indicate significant differences according to one-way ANOVA followed by Duncan’s multiple range test (*p* < 0.05); treatments sharing at least one letter are not significantly different. On day 0, the single letter “a” applies collectively to all five treatments because no significant differences were detected among them.

The temporal changes in available phosphorus, available potassium, and total nitrogen concentrations are presented in Figure 4. In T1 and T2, available phosphorus and available potassium changed only slightly during the first 15 days (Figure 4a,b). In T5, available phosphorus increased from 17.97 g/kg on day 0 to 21.95 g/kg on day 15, corresponding to a descriptive increase of 22.15%. Available potassium increased from 9.26 g/kg to 12.34 g/kg over the same period, corresponding to a descriptive increase of 33.26%. On day 15, the concentrations of both nutrients in T5 were significantly higher than the corresponding concentrations in all other treatments (*p* < 0.05). The total nitrogen concentration in T5 was 12.99 g/kg on day 0 and 16.12 g/kg on day 15.

### 3.6. Seed Bioassay Indicators and Ascaris Egg Mortality

**Figure 5:**
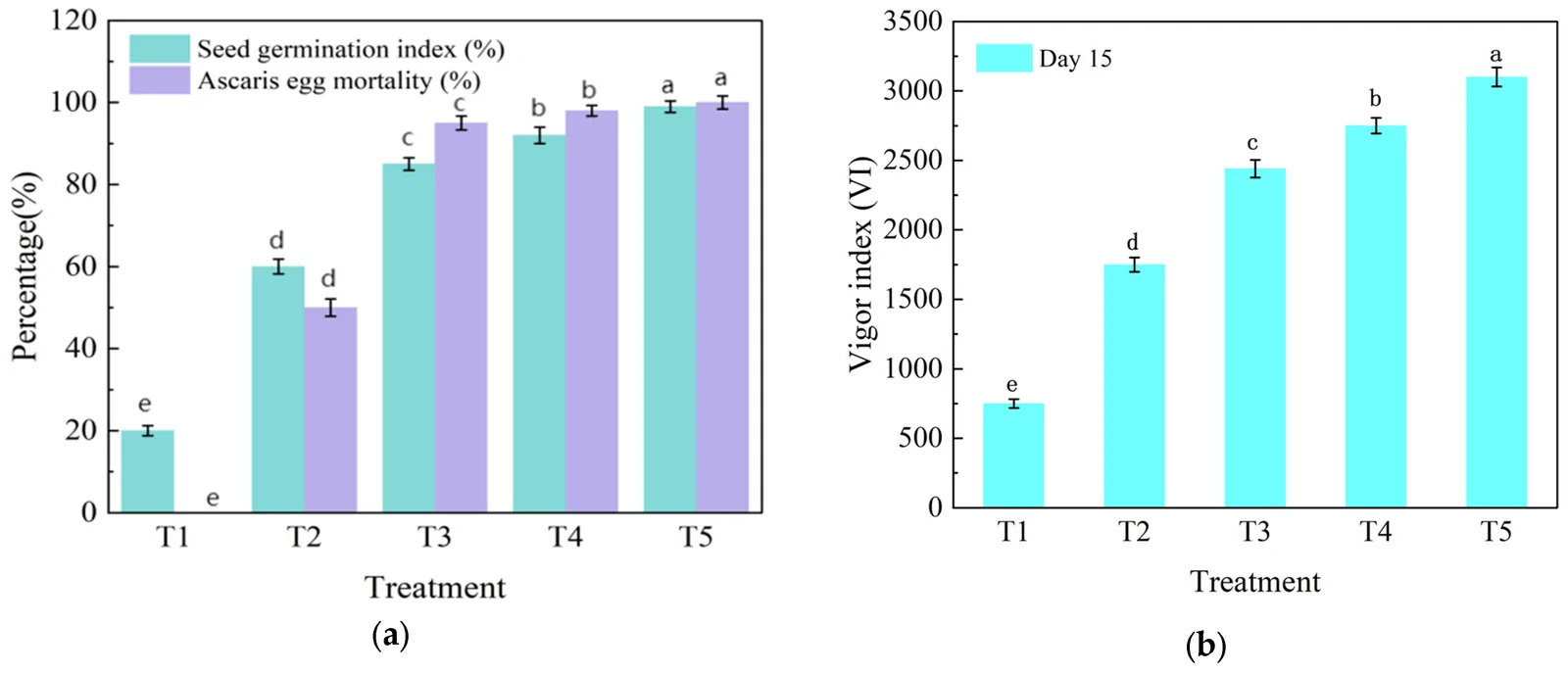
Seed bioassay and Ascaris egg mortality outcomes after 15 days of composting: (**a**) germination index (GI) and *Ascaris* egg mortality; (**b**) vigor index (VI), calculated using mean seedling dry mass. Data are presented as mean ± SD of three independently prepared compost piles (*n* = 3). Different lowercase letters indicate significant differences among treatments according to one-way ANOVA followed by Duncan’s multiple range test (*p* < 0.05); treatments sharing at least one letter are not significantly different. Letters were assigned separately within each endpoint.

The seed germination index is widely used to evaluate compost maturity and phytotoxicity [24]. As shown in Figure 5a, T1 had a GI of only 20% on day 15, and no mortality of Ascaris eggs was observed. The GI of T2 increased to 60%, whereas Ascaris egg mortality reached 50%. The greatest improvement was recorded in T5, which achieved a GI of 99% and 100% Ascaris egg mortality. The 100% Ascaris egg mortality observed in T5 met the requirement of ≥95% specified for organic fertilizers in NY/T 525-2021 [28]. This comparison refers specifically to the Ascaris egg mortality criterion and does not constitute a comprehensive assessment of compost biosafety. The seed vigor index combines the germination index with seedling dry mass and therefore provides an additional measure of seedling performance under the bioassay conditions [37]. As presented in Figure 5b, T1 had a VI of only 750, indicating severe phytotoxicity. The VI of T2 increased to 1750, reflecting a reduction in toxicity, whereas T5 produced the highest value of 3100.

### 3.7. Temporal Dynamics of Enzyme Activities During Chicken Manure Composting

**Figure 6:**
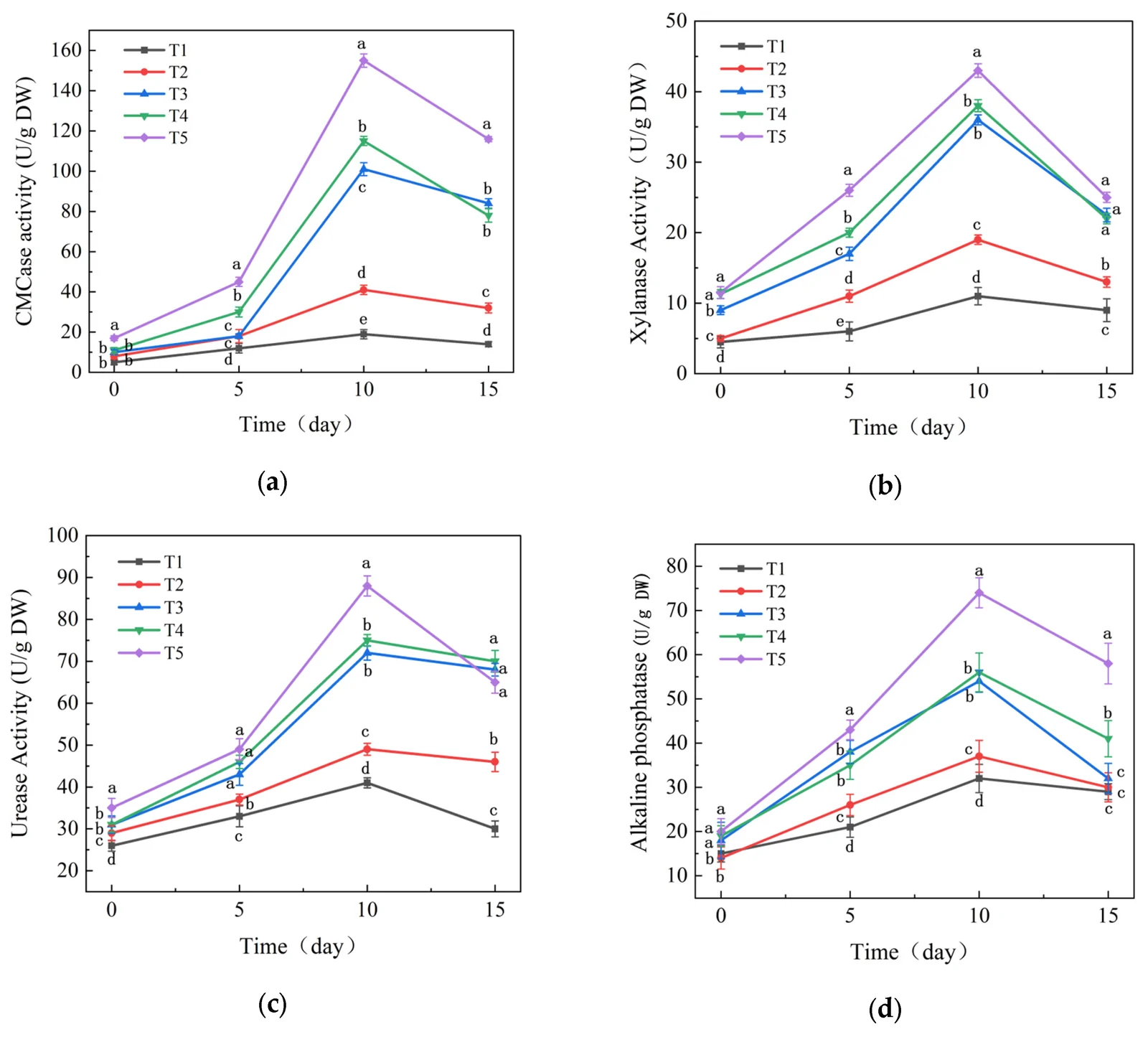
Enzyme activities during the first 15 days of composting: (**a**) carboxymethyl cellulase (CMCase); (**b**) xylanase; (**c**) urease; and (**d**) the reported alkaline phosphatase endpoint. Activities are expressed as enzyme units per gram of dry weight (U g^−1^ DW). Data are presented as mean ± SD of three independently prepared compost piles (*n* = 3). Different lowercase letters at the same sampling time indicate significant differences according to one-way ANOVA followed by Duncan’s multiple range test (*p* < 0.05); treatments sharing at least one letter are not significantly different.

Microbial enzymes mediate many of the biochemical transformations that stabilize organic matter during composting [38]. Across the first 15 days, cellulase, xylanase, urease, and alkaline phosphatase generally increased to day 10 and then declined, although their absolute activities differed among treatments (Figure 6). In T5, cellulase rose from 45 U g^−1^ DW on day 5 to 155 U g^−1^ DW on day 10 before decreasing to 116 U g^−1^ DW on day 15. On day 10, cellulase activities in T3 and T2 were 101 and 24 U g^−1^ DW, respectively (Figure 6a). Xylanase in T5 increased from 26 U g^−1^ DW on day 5 to 43 U g^−1^ DW on day 10; at the same time point, T4, T3, T2, and T1 showed activities of 38, 36, 13, and 11 U g^−1^ DW, respectively (Figure 6b). Urease activity in T5 peaked at 88 U g^−1^ DW on day 10, whereas T4, T3, T2, and T1 reached 75, 72, 49, and 41 U g^−1^ DW, respectively. By day 15, urease activity had declined to 65 U g^−1^ DW in T5, 46 U g^−1^ DW in T2, and 30 U g^−1^ DW in T1 (Figure 6c). Alkaline phosphatase likewise peaked on day 10; T5 showed the highest reported activity (74 U g^−1^ DW) and retained an activity of 58 U g^−1^ DW on day 15 (Figure 6d).

## 4. Discussion

### 4.1. Responses to the Inoculant and Bulking Agent Formulations

The rate at which a compost pile enters the thermophilic phase and the duration of elevated temperatures are important indicators of composting performance and hygienic treatment [38]. The high moisture content and limited porosity of chicken manure can restrict aeration and indigenous microbial activity [39]. Consistent with this, T1 showed only limited heating. T2, which contained the bulking agents, reached a maximum temperature of 46 °C but showed a less pronounced thermal response than T3–T5. The moderate heating observed in T2 is consistent with improved matrix conditions, possibly through increased porosity or improved moisture distribution. However, these physical properties were not measured directly.

T3 and T4 also exhibited pronounced heating, indicating strong thermal responses to both the commercial inoculant and the inoculant prepared in this study. T5 nevertheless showed the strongest and most sustained thermal response. It reached 74 °C on days 5 and 10 and remained above 50 °C through day 30 at the plotted sampling times. Relative to T2 and T4, the stronger response of T5 is consistent with complementary effects of microbial inoculation and matrix amendment [40]. However, the present statistical analysis did not estimate a formal interaction term.

The enzyme activity patterns provide a possible biological explanation for the thermal response. On day 5, T5 exhibited cellulase and xylanase activities of 45 and 26.0 U/g DW, respectively, compared with approximately 13 U/g DW for both enzymes in T2. Hydrolytic enzymes may facilitate lignocellulose depolymerization [41], thereby increasing the availability of substrates for microbial metabolism and associated heat production [42]. Because these activities were measured in composite samples from each pile, they probably reflected the combined responses of the introduced and indigenous microorganisms.

The stronger enzyme and temperature responses in T5 than in T4 suggest that the bulking agent matrix may have favored the performance of the composite inoculant under the tested conditions. Changes in matrix structure could facilitate oxygen transfer, substrate contact, and extracellular enzyme activity. Together, the temperature and enzyme activity patterns suggest that the amendments altered the matrix and increased hydrolytic activity. These changes may have jointly contributed to rapid heating and to the transformation of organic matter during the early stages of composting. As decomposition progressed, changes in substrate availability may have further supported microbial activity, forming a possible positive feedback process [43]. The combined formulation is therefore notable for coordinated gains in both thermal performance and hydrolytic activity, rather than for improvement in any single composting measure.

### 4.2. Interpreting C/N During the Early Stages of Lignocellulosic Composting

Compost maturity and suitability for agricultural use involve phytotoxicity, hygienic quality, and biochemical stability and therefore cannot be represented fully by a single index [44]. Within 15 days, T5 achieved a germination index of 99% and 100% mortality of the assayed Ascaris eggs, while its operational C/N ratio reached 8.00 on day 15. These endpoints should nevertheless be interpreted separately because they characterize different properties of the compost. Accordingly, the seed bioassay specifically indicates the phytotoxicity of water-extractable compounds under the defined assay conditions. In contrast, the Ascaris egg assay evaluates a targeted hygiene outcome, whereas the C/N ratio characterizes the relative proportions of organic carbon and total nitrogen.

The operational C/N ratio of T5 showed an overall decrease from approximately 8.9 on day 0 to 8.0 on day 15. On day 15, T5 (8.00) did not differ significantly from T3 (8.20), but it was significantly lower than T1 (8.50) and T2 (8.65) and significantly higher than T4 (7.80) (*p* < 0.05). These treatment differences may partly reflect variation in the composition and degradability of the composting materials. Readily degradable compounds in the manure may have been transformed more rapidly than the structural carbon supplied by corn straw and the other bulking agents of plant origin. The elevated cellulase and xylanase activities in T5 are consistent with active lignocellulose hydrolysis, although the present data do not establish a direct mechanistic relationship between enzyme activity and the change in C/N. Overall, the observed C/N pattern is compatible with ongoing organic-matter transformation, but it does not by itself determine the extent or underlying processes of decomposition.

The high germination index suggests low phytotoxicity in aqueous extracts under the stated bioassay conditions rather than complete detoxification of the compost. Likewise, the higher total nitrogen concentration is compatible with nitrogen retention, although concentration resulting from dry matter loss may also have contributed. A mass balance for the entire pile would be required to distinguish the relative contributions of these processes.

C/N should therefore be interpreted together with germination response, temperature history, and complementary stability and hygiene indicators [43]. On day 15, the germination index provided biological evidence that complemented, rather than replaced, the compositional information represented by C/N. More broadly, this interpretation emphasizes that compost-maturity indicators should be judged in the context of feedstock composition and the duration of processing. Additional curing, accompanied by direct stability measurements, would better define the characteristics and persistence of the residual organic carbon.

### 4.3. Potential Relationships Among Enzyme Activities, Odor Control, and Nutrient Availability

Reducing odor emissions while maintaining nutrients in forms available to plants is important for the environmental and agricultural performance of chicken manure composting [43]. During composting, the transformation of compounds containing nitrogen or sulfur can result in the formation and volatilization of ammonia and hydrogen sulfide [35]. On day 15, T5 showed the largest decrease in the measured VOC endpoint. Relative to day 0, its measured ammonia and hydrogen sulfide concentrations decreased by 96.95% and 99.90%, respectively. Available phosphorus, available potassium, and total nitrogen concentrations also increased during this period. These changes show that lower concentrations of odorous compounds coincided with higher nutrient concentrations in the combined formulation.

The gas measurements represent concentrations at the specified sampling times rather than cumulative emissions from the entire pile. They therefore characterize changes in the sampled composting environment but do not quantify total gaseous losses during the experiment.

The urease activity pattern provides one possible explanation for the nitrogen response. T5 reached a peak urease activity of 88 U/g DW without a corresponding increase in the measured ammonia concentration. Ammonium released through urea hydrolysis may have been assimilated by microorganisms or retained in less volatile forms within the amended matrix [45]. Temperature, moisture, pH, gas transfer, and indigenous microbial activity could also have influenced the measured ammonia concentration. The transformation of precursors containing sulfur, together with changes in volatile organic compounds, may partly explain the lower H_2_S concentration and VOC endpoint [46].

Enhanced enzyme activities also occurred alongside increases in available phosphorus and available potassium [43]. The 22.15% increase in available phosphorus may reflect a combination of microbial and physicochemical solubilization processes. Hydrolysis of organic phosphorus by phosphatases may release inorganic phosphate [47], although its contribution cannot be resolved from the current measurements. Similarly, decomposition of plant residues, potentially facilitated by cellulase and xylanase activity, may have promoted the release of soluble potassium and contributed to the 33.26% increase in available potassium.

The ability of the composite inoculant to solubilize phosphorus and potassium in vitro supports its functional potential. Nevertheless, nutrient concentrations in the compost piles were also influenced by substrate decomposition, microbial assimilation, pH, mineral equilibria, and dry matter loss. Taken together, the findings are consistent with combined contributions from enzymatic hydrolysis, microbial transformation, substrate release, and physicochemical processes. Overall, these responses suggest that coupling microbial inoculation with matrix amendment may enhance odor-related indicators and nutrient availability during the first 15 days of composting, although the relative contributions of the underlying processes remain unresolved.

### 4.4. Experimental Scope, Key Constraints, and Research Priorities

This experiment was designed primarily to assess the formulation during early composting. Each treatment comprised three independently prepared piles with an initial mass of 50 kg. Temperature was monitored through day 45, whereas most chemical, enzymatic, and biological measurements were restricted to the first 15 days. Interpretation should therefore focus on short-term responses under the tested conditions rather than on complete compost maturation. Within this defined period, T5 showed favorable responses across multiple thermal, enzymatic, odor-related, nutrient, and bioassay endpoints.

The comparative treatment design was not intended to resolve the separate effects of the viable inoculant, the amended substrate matrix, and the indigenous microbial community. Mechanistic follow-up experiments should therefore monitor the introduced strains individually, characterize temporal shifts in community composition, and incorporate controls containing inactivated inoculum or sterilized amendments, together with an appropriate non-solubilizing negative reference. These additions would permit a more direct assessment of the contribution of each component. Moreover, because characterization of the commercial inoculant relied on information supplied by the manufacturer, T3 is best regarded as a benchmark for comparison between complete product formulations rather than between defined microbial strains.

The pak choi seed and Ascaris egg assays addressed two specific outcomes: phytotoxicity of aqueous compost extracts and mortality of the tested parasite eggs, respectively. Broader ecotoxicological endpoints and additional pathogen assessments would strengthen the evaluation of compost safety. Longer curing and storage periods, together with whole-pile mass balances, are needed to establish stability and nutrient retention. Finally, larger-scale composting trials and field experiments are required to determine whether the short-term responses observed here translate into sustained agronomic benefits.

## 5. Conclusions

This study evaluated a multifunctional composite microbial inoculant comprising *K. rosea* ACCC 10488, *B. subtilis* ACCC 60364, *T. longibrachiatum* ACCC 32584, and *L. plantarum *ACCC 11118. The four strains showed in vitro compatibility and distinct functional activities potentially relevant to organic matter degradation and nutrient mobilization. Under the tested conditions, combining the inoculant with agricultural waste bulking agents was associated with favorable changes in several measured composting endpoints during the first 15 days. The combined formulation (T5) showed more rapid and sustained pile heating, a favorable change in the reported VOC endpoint, and lower measured ammonia and hydrogen sulfide concentrations. T5 also exhibited higher selected hydrolytic enzyme activities than T2 at several sampling times. By day 15, its available phosphorus, available potassium, and total nitrogen concentrations were higher than their respective initial values. T5 additionally achieved a pak choi seed germination index of 99% and 100% mortality of the assayed Ascaris eggs. Together, the treatment comparisons support further evaluation of the combined formulation for improving selected indicators of chicken manure composting during its early stages. The microbial basis of this response, including strain persistence and any formal interaction between the inoculant and bulking agents, remains to be clarified. Most chemical and biological endpoints were assessed during the first 15 days using three independent piles per treatment. Studies with longer curing periods and trials conducted at a greater scale are therefore needed to confirm sustained performance. Monitoring at the strain level, microbial community and functional analyses, and field validation would further clarify the underlying biological processes and assess compost stability and agronomic performance over longer periods.

## Figures and Tables

**Table 1:** Physicochemical characteristics of the raw materials used in the composting treatments.

Parameter	Chicken Manure	Corn Straw	Furfural Residue	Plant Ash
Organic matter (%)	76.81 ± 0.54	93.88 ± 0.67	71.34 ± 0.52	<0.1 *
Total nitrogen (%)	5.63 ± 0.20	0.60 ± 0.06	0.45 ± 0.03	<0.1 *
Total phosphorus (%)	4.19 ± 0.15	0.27 ± 0.03	0.60 ± 0.04	2.40 ± 0.10
Total potassium (K_2_O, %)	1.56 ± 0.06	2.28 ± 0.05	1.10 ± 0.03	8.02 ± 0.15
Total nutrients (%)	11.38 ± 0.26	3.15 ± 0.08	2.15 ± 0.06	10.42 ± 0.18
Humic acid (%)	4.87 ± 0.17	46.23 ± 0.20	16.43 ± 0.41	<0.1 *

Note: Values are mean ± standard deviation (SD) of three analytical determinations of one homogenized sample of each raw material (*n* = 3 technical replicates). These replicates describe analytical precision and are not independent batches of raw material. All composition values are expressed on a dry mass basis. * Values reported as <0.1% were below the laboratory reporting limit of 0.1% and were not used as quantitative concentrations.

**Table 2:** In vitro phosphate and potassium solubilization by the individual strains and the composite culture. Values are mean ± SD of three replicate assays (*n* = 3). D/d is the ratio of dissolution zone diameter (D) to colony diameter (d). Different lowercase superscript letters within the same response column indicate significant differences according to one-way ANOVA followed by Duncan’s multiple range test (*p* < 0.05). Letters were assigned independently within each column; means sharing at least one letter were not significantly different.

Culture	Dissolution Zone Diameter, D (mm)	Colony Diameter, d (mm)	D/d Ratio	Potassium Solubilization Efficiency (%)
*K. rosea*	72.4 ± 1.8 ^c^	22.0 ± 0.7 ^b^	3.2 ± 0.1 ^c^	83.4 ± 1.6 ^c^
*B. subtilis*	73.5 ± 2.0 ^c^	17.0 ± 0.6 ^c^	4.3 ± 0.3 ^b^	82.5 ± 1.3 ^c^
*T. longibrachiatum*	77.4 ± 2.2 ^b^	11.7 ± 0.5 ^d^	6.6 ± 0.2 ^a^	87.6 ± 1.7 ^b^
*L. plantarum*	72.4 ± 1.7 ^c^	10.7 ± 0.4 ^d^	6.7 ± 0.3 ^a^	82.9 ± 1.4 ^c^
Composite culture	92.4 ± 2.5 ^a^	25.9 ± 0.9 ^a^	3.5 ± 0.1 ^c^	96.5 ± 1.1 ^a^

## Data Availability

The original contributions presented in this study are included in the article/Supplementary Material. Further inquiries can be directed to the corresponding authors.

## Supplementary Materials

The following supporting information can be downloaded at: https://www.mdpi.com/article/10.3390/microorganisms14092052/s1, Figure S1: Cross-streak compatibility assay for the four strains used in the composite inoculant.
