# Supplementary material for: Combined Application of a Composite Microbial Inoculant and Bulking Agents Improves Chicken Manure Composting
Source: Microorganisms. 2026 Sep 14;14(9):2052. doi: 10.3390/microorganisms14092052 (PMC13609110; doi:10.3390/microorganisms14092052)
Supplement: Supplementary file 1 [file microorganisms-14-02052-s001.zip › microorganisms-4514455-supplementary.pdf]

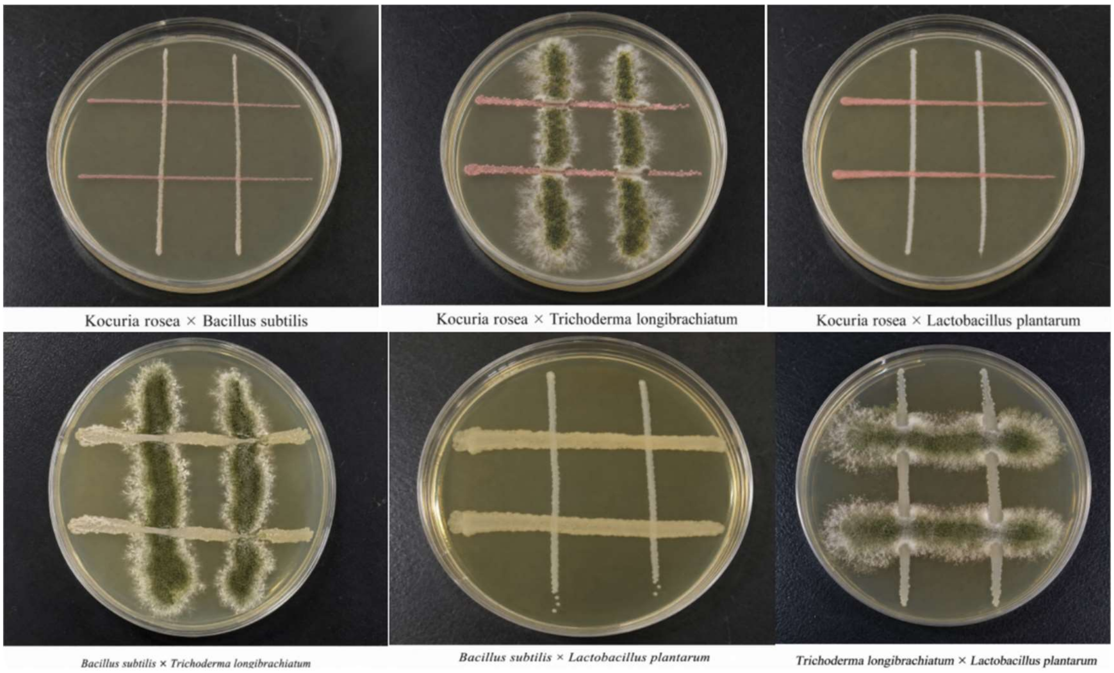

Supplementary Figure S1. Cross-streak compatibility assay for the four strains used in the composite inoculant. Biocompatibility of all pairwise strain combinations was determined via the cross-streak assay on TSA plates at 28 °C. Panels 1–6 correspond to the six pairwise combinations of *Kocuria rosea, Bacillus subtilis, Trichoderma longibrachiatum,* and *Lactobacillus plantarum*. All strains grew normally at the crossing sites without obvious antagonistic zones, confirming favorable compatibility for composite microbial inoculant preparation.
